# Bark Beetle-Associated Blue-Stain Fungi Increase Antioxidant Enzyme Activities and Monoterpene Concentrations in *Pinus yunnanensis*

**DOI:** 10.3389/fpls.2018.01731

**Published:** 2018-11-27

**Authors:** Yue Pan, Tao Zhao, Paal Krokene, Ze-fen Yu, Min Qiao, Jun Lu, Peng Chen, Hui Ye

**Affiliations:** ^1^Laboratory of Biological Invasion and Ecosecurity, Yunnan University, Kunming, China; ^2^Eco-Development Academy, Southwest Forestry University, Kunming, China; ^3^Department of Science and Technology, Örebro University, Örebro, Sweden; ^4^Norwegian Institute of Bioeconomy Research, Ås, Norway; ^5^Laboratory for Conservation and Utilization of Bio-Resources, Key Laboratory Resources of the Ministry of Education, Yunnan University, Kunming, China; ^6^Yunnan Academy of Forestry, Kunming, China; ^7^Laboratory of Ecology and Evolutionary Biology, Yunnan University, Kunming, China

**Keywords:** *Leptographium wushanense*, *L. sinense*, *Ophiostoma canum*, phloem reaction zone, defense response

## Abstract

Yunnan pine is the most important tree species in SW China in both economical and ecological terms, but it is often killed by pine shoot beetles (*Tomicus* spp.). *Tomicus* beetles are secondary pests in temperate regions and the aggressiveness of the beetles in SW China is considered to be due to the warm subtropical climates as well as the beetles’ virulent fungal associates. Here, we assessed the virulence of three blue-stain fungi (*Leptographium wushanense*, *L. sinense* and *Ophiostoma canum*) associated with pine shoot beetles to Yunnan pine (*Pinus yunnanensis*) in SW China. Following fungal inoculation, we measured necrotic lesion lengths, antioxidant enzyme activities and monoterpene concentrations in the stem phloem of Yunnan pine. *Leptographium wushanense* induced twice as long lesions as *L. sinense* and *O. canum*, and all three fungi induced significantly longer lesions than sterile agar control inoculations. The activity of three tested antioxidant enzymes (peroxidase, polyphenol oxidase, and superoxide dismutase) increased after both fungal inoculation and control inoculation. However, *L. wushanense* and *L. sinense* generally caused a greater increase in enzyme activities than *O. canum* and the control treatment. Fungal inoculation induced stronger increases in six major monoterpenes than the control treatment, but the difference was significant only for some fungus-monoterpene combinations. Overall, our results show that *L. wushanense* and *L. sinense* elicit stronger defense responses and thus are more virulent to Yunnan pine than *O. canum*. The two *Leptographium* species may thus contribute to the aggressiveness of their beetle vectors and could damage Yunnan pine across SW China if they spread from the restricted geographical area they have been found in so far.

## Introduction

Conifers and bark beetles in the subfamily Scolytinae have developed intricate relationships over millions of years of interaction and co-evolution. Conifers have evolved multi-purpose constitutive and inducible defenses to fend off attackers, whereas bark beetles have evolved different strategies to overcome these defenses (Adams et al., 2011; Krokene, 2015). One beetle strategy is to vector diverse phytopathogenic blue-stain fungi of varying virulence that infect the tree when the beetles tunnel in the phloem (Zhou et al., 2002; Masuya et al., 2003). These fungi are thought to facilitate bark beetle host colonization (Krokene, 2015). Blue-stain fungi are necrotrophic pathogens that gradually colonize phloem and xylem tissues away from the beetle tunnels, capturing tree resources (Six and Wingfield, 2011; Six, 2012), eliciting tree defenses (Franceschi et al., 2000; Nagy et al., 2000; Viiri et al., 2001; Lieutier et al., 2009; Zhao et al., 2010, 2011a,b), and likely helping bark beetles overwhelm trees defenses (Paine et al., 1997; Lieutier et al., 2009; Zhao et al., 2018). In addition, some blue-stain fungi appear to be involved in nutritional supplementation of bark beetle larvae (Ayres et al., 2000; Bleiker and Six, 2007) and production of bark beetle aggregation pheromones (Zhao et al., 2015).

Conifer defense reactions are complex and involve several metabolic pathways, such as the isoprenoid and phenylpropanoid pathways (Iriti and Faoro, 2009). Pines and other members of the family Pinaceae rely heavily on monoterpenes and other products of the isoprenoid pathway in their defense against bark beetles and other biotic challenges (Franceschi et al., 2000; Nagy et al., 2000; Viiri et al., 2001; Klepzig et al., 2005; Fäldt et al., 2006; Zhao et al., 2011a). Monoterpenes stored constitutively in resin ducts in the phloem and sapwood are the first defense barrier (Gershenzon and Dudareva, 2007). Following beetle attack and fungal infection, monoterpene concentrations increase dramatically in the necrotic lesions formed by the trees’ induced defenses (e.g., Viiri et al., 2001; Klepzig et al., 2005; Fäldt et al., 2006; Zhao et al., 2011a). Since more virulent fungi induce longer necrotic lesions and stronger monoterpene responses upon infection than less virulent fungi (Krokene and Solheim, 1999; Fäldt et al., 2006), fungal virulence is often assessed by measuring lesion lengths and quantifying monoterpene increases following experimental inoculation.

In addition to terpenes, pines and other plants protect themselves against damage by releasing antioxidative scavenging enzymes such as superoxide dismutases (SOD), polyphenol oxidases (PPO), peroxidases (POD), and catalases (CAT). These enzymes protect plants from reactive oxygen species that are released when plant tissues are damaged by feeding insects colonizing fungi (Liao, 2002; Liu, 2010). Previous studies have demonstrated that salt and drought stress, wounding, beetle attack and fungal infection may alter the activity of these enzymes in plants (Nagy et al., 2004; Pan et al., 2006). However, little is known about antioxidative enzymes in conifers and if they are involved in tree resistance and tolerance against bark beetles and blue-stain fungi (Liao, 2002; Liu, 2010).

Yunnan pine (*Pinus yunnanensis*) is an economically and ecologically important conifer in SW China that is suffering large-scale damage from pine shoot beetles in the genus *Tomicus* (Scolytinae) (Ye, 1992; Lu, 2011). Since the 1980’s, 93,000 ha of *P. yunnanensis* has been killed by three native *Tomicus* species: *T. yunnanensis*, *T. minor* and *T. brevipilosus* (Ji et al., 2007; Lu, 2011). In Europe, *Tomicus* species are usually secondary pests which can only breed in the stem phloem of weakened or dead trees, but their maturation feeding in the shoots of healthy trees may cause substantial increment losses (Jankowiak and Kurek, 2006; Jankowiak, 2008). However, *Tomicus* species may behave aggressively and kill healthy individuals of their co-evolved host tree Yunnan pine (Lu, 2011). Virulent fungal associates, together with warm and humid climates, are thought to be the explanation for the beetles’ aggressiveness in the area (Duan, 1999).

*Tomicus* is associated with many different blue-stain fungi in SW China (Zhou et al., 2000, 2013; Paciura et al., 2010; Chang et al., 2017; Pan et al., 2017, 2018) and inoculation studies have demonstrated that at least one of them, *L. yunnanense*, is virulent to Yunnan pine (Liao and Ye, 2002). Recently, we isolated three species of blue-stain fungi with unknown virulence from *Tomicus* breeding galleries: *Leptographium wusanense, L. sinense,* and *Ophiostoma canum*. The objective of this study was to determine if these fungi are virulent to Yunnan pine and assess their potential damage to trees. To this end, we inoculated the fungi into the phloem of mature trees and quantified tree defense responses such as lesion lengths, antioxidative enzyme activities, and monoterpene concentrations.

## Materials and Methods

### Fungal Species and DNA Sequencing

Three species of blue-stain fungi associated with pine shoot beetles (*Tomicus* spp.) in China were included in this study (Table 1). *Leptographium sinense* and the newly described species *L. wusanense* were isolated from galleries of *T. armandii* infesting Armand pine (*P. armandii*). *Ophiostoma canum* was isolated from a gallery of *T. yunanensis* in Yunnan pine. Armand pine is usually relatively resistant to *Tomicus* (Zhao and Längström, 2012), but since Armand pine and Yunnan pine often forms mixed stands, blue-stain fungi may be transferred between the two species by *Tomicus* beetles. *Leptographium sinense* has previously been isolated from *Hylobitelus xiaoi*, a weevil causing serious damage to several pine species in China (Yin et al., 2015). The third fungal species, *Ophiostoma canum*, is a common associate of *Tomicus* in Yunnan pine in China (Pan et al. manuscript).

**Table 1:** Isolates of blue-stain fungi used in this study and GenBank accession numbers for DNA sequences. *L., Leptographium; O., Ophiostoma.*

Fungus	Isolate no.	Host/insect vector	Origin	GenBank accession no.			
				ITS1-5.8S-ITS2	ITS2-LSU	β -tubulin	EF-1 α
*L. wushanense*	YMF 1. 04934	*Pinus armandii*/*Tomicus armandii*	Wushan,Chongqin, China	______	MG878407	MG878408	MG878409
*L. sinense*	YMF 1. 04935	*P. armandii*/*T. armandii*	Liupanshui, Guizhou, China	______	MH216027	MH216030	MH216033
*O. canum*	YMF 1. 04936	*P. yunnanensis*/*T. yunnanensis*	Luliang,Yunnan, China	MG702035	_______	MG702062	MG702089


The three blue-stain fungi were identified based on morphology and sequencing of three DNA regions (for details see Supplementary Figures S1, S2). DNA was extracted as described in Raeder and Broda (1985). For all three species we amplified partial sequences of β-tubulin using the primer pair Bt2a/Bt2b, and elongation factor 1α (EF-1α) using EF1F/EF2R (Glass and Donaldson, 1995; Jacobs et al., 2004). Furthermore, for *L. sinense* and *L. wushanense* we amplified the internal transcribed spacer 2 region and part of the large subunit (ITS2-LSU), using the primer pair ITS3/LRS (Vilgalys and Hester, 1990; White et al., 1990). For *O. canum*, we amplified the ITS1 and 2 regions including the 5.8S gene (ITS1-5.8S-ITS2), using the primer pair ITS1F/ITS4 (White et al., 1990; Gardes and Bruns, 1993). All PCR conditions were according to the procedure described by Grobbelaar et al. (2009).

The obtained DNA sequences were compared with relevant *Leptographium* and *Ophiostoma* sequences deposited in GenBank. All sequences were then aligned using ClustalX1.81 (Jeanmougin et al., 1998). Data for the three sequenced DNA regions (ribosomal DNA, β-tubulin and EF-1α) were combined using BioEdit 7.0.1. Phylogenetic relationships were determined based on Bayesian (BI) analysis using MrBayes 3.1.2 (Ronquist and Huelsenbeck, 2003) and Maximum likelihood (ML) analysis using RAxML version 7.0.4 (Stamatakis, 2014). Two phylogenetic trees clustered our sequenced *L. sinense* and *O. canum* strains together with other *L. sinense* and *O. canum* sequences from GenBank with high support (93 and 100% Bayesian posterior probabilities (BIPP), respectively, Supplementary Figures S1, S2). The *Leptographium* tree placed *L. wushanense* in the *L. lundbergii* complex, as a sister taxon of *L. conjunctum* and *L. yunnanense* with 99% BIPP.

The fungal strains used in this study were deposited in the Herbarium of the Laboratory for Conservation and Utilization of Bio-resources, Yunnan University, Kunming, Yunnan, China (YMF; formerly Key Laboratory of Industrial Microbiology and Fermentation Technology of Yunnan). Sequence data were deposited in GenBank (accession numbers are provided in Table 1).

### Field Procedures

The experiment was carried out in a naturally regenerated Yunnan pine forest in Yunnan, SW China (25°12′N, 102°77′E) in July and August 2016. Twenty to thirty years old Yunnan pines with no apparent damage and of similar size (diameter at breast height: 13 ± 3 cm, height: 15 ± 3 m (mean ± SD) were selected for inoculation. Each tree was inoculated three times with each fungus and three times with the control using a 1 cm diameter cork borer. Inoculum consisted of fungal cultures (Table 1) incubated on 2% malt extract agar for 14 days at 20°C in the dark or sterile malt extract agar as a control. The 12 inoculation sites on each tree were positioned in a spiraling pattern around the stem from 0.7 to 1.7 m height, with alternating positions for each of the four treatments. Lesion lengths induced by the different inoculation treatments were recorded 10, 20, and 30 days after inoculation by carefully removing the cork bark over one set of inoculation holes and measuring the full length of the necrotic lesions in the phloem (one inoculation site per inoculation treatment on each sampling day). At the same time, a phloem sample was taken from each necrotic lesion using a sterilized scalpel and forceps. The phloem samples collected each day were evenly assigned to two groups. One group was used for terpene analysis and another group for determining antioxidative enzyme activity. After sampling, phloem samples were placed individually in 1.5 mL sterilized Eppendorf tubes and immediately stored in liquid nitrogen. Two or three additional phloem samples (12 ± 3 mm × 1.5 ± 0.5 mm) were taken from each necrotic lesion and cultured on 2% malt extract agar to reisolate the inoculated fungi.

### Determination of Antioxidative Enzyme Activity

Four antioxidative enzymes (CAT, POD, PPO, SOD) were extracted from control and fungus inoculated phloem samples collected 10, 20, and 30 days after inoculation (*n* = 10 trees). Enzyme activity was determined using specific kits following the manufacturer’s instructions (Suzhou Comin Biotechnology Co., Ltd., Suzhou, Jiangsu, China). The specific determination method used for each of the four enzymes is given in Supplementary Table S1. Briefly, phloem was ground into a fine power under liquid nitrogen, 0.1 g powder was added to 1 mL reagent (Supplementary Table S1) in an Eppendorf tube and placed in an ice bath. The supernatant was then transferred to a new Eppendorf tube and centrifuged at 8000 rpm for 10 min at 25°C. The resulting crude enzyme extract was mixed with defined reagents following the instructions outlined in Supplementary Table S1. The absorbance of each post-reaction solution was measured using an ultraviolet visible spectrophotometer (model 752N, Shanghai analytical instrument factory, Shanghai, China) and the activity of each antioxidative enzyme was calculated according to the specific formulas given in Supplementary Table S1.

One unit of SOD activity was defined as the amount of enzyme required to cause a 50% inhibition in the reduction of Nitro Blue Tetrazolium (NBT). One unit of POD and PPO activity was defined as the amount of enzyme required to cause a 0.01 change in absorbance at 525 (PPO) and 470 (POD) nm per minute. One unit of CAT activity was defined as the amount of enzyme required to decompose 1 nmol H_2_O_2_ at 240 nm per minute.

### Monoterpene Separation, Identification, and Quantification

All phloem samples from 10 of the 20 experimental trees were submerged individually in 1.5 mL hexane in 8 mL brown sampling vials (Aijiren, China) and kept at 20°C for 48 h. The extract was centrifuged at 5000 rpm for 10 min at 8°C, 1 mL supernatant was transferred to a 2 mL sampling vial (Agilent, United States), and 0.3 mg nonene (99%, donated by Dr. Ehrenstorfer) was added as an internal standard. Extracts were stored at -20°C until gas chromatography-mass spectrometry (GC-MS) analysis. The remaining phloem was dried at 80°C for 6 h and weighted on an electronic balance (Sartorius) to calculate monoterpene concentrations per unit dry weight.

An Agilent 6890 GC connected to a 5973 MS and a HP-5 fused silica capillary column (J&W Scientific^TM^, 30 m length, 0.25 mm inner diameter and 0.25 μm film thickness) was used for the analysis. One microliter extract was injected to a split/splitless injector with a 30 s splitless injection at a temperature of 250°C. The temperature program started at 40°C, increased to 80°C at a rate of 3°C/min, then to 280°C at a rate of 5°C/min, and then remained at 280°C for 20 min.

Monoterpenes were identified using the Wiley 7 n.l and NIST 98.L reference libraries and confirmed by comparing retention times and mass spectra with available authenticated standards. The absolute amount of each monoterpene (m_x_) was calculated using the equation m_x_ = k(A_x_/A_s_) m_s_/D_w_ (where k is the relative response factor, A_x_ is the peak area of a monoterpene in the sample, A_s_ is the peak area of the internal standard nonane, m_s_ is the mass of nonane and D_w_ is the dry weight of the sample). The relative response factor (k) was calculated using the equation k = A_s_m_i_/(A_i_m_s_) (where A_s_ is the peak area of nonane, A_i_ is the peak area of a monoterpene standard, m_s_ is the mass of nonane, and m_i_ is the mass of the monoterpene standard).

### Statistical Analyses

All analyses were conducted with SPSS 17.0 (SPSS Inc., Chicago, IL, United States), and graphs were generated using Origin 8.5 (Origin Lab Corporation, Northampton, MA, United States). The variation in lesion lengths was analyzed using repeated measures model, and enzymes activities and monoterpene concentrations was tested using a linear mixed model (GLMM) for repeated measures since we had some missing data caused by a few samples that were too diluted for enzyme and monoterpene analysis. Following ANOVA, differences between inoculation treatments at each time point and between time points for each treatment were tested using the Bonferroni test (for data with equal variances) or Dunnett’s T3 test (for data with unequal variances) at *P* = 0.05.

## Results

### Phloem Lesion Length

All fungi were pathogenic to Yunnan pine and induced significantly longer lesions than agar control inoculations at all three time points [10^th^ day: *F*_(3,__39.796)_ = 30.321, *P* < 0.001; 20^th^ day: *F*_(3,__40.870)_ = 72.155, *P* < 0.001; 30^th^ day: *F*_(3,__39.635)_ = 86.683, *P* < 0.001]. The three fungi showed different virulence to Yunnan pine. *Leptographium wushanense* induced very long lesions (up to 103 mm) that were significantly longer than those induced by *L. sinense* and *O. canum* at all three time points. *Leptographium sinense* induced slightly but significantly longer lesions than *O. canum* 20 and 30 days after inoculation.

**FIGURE 1:**
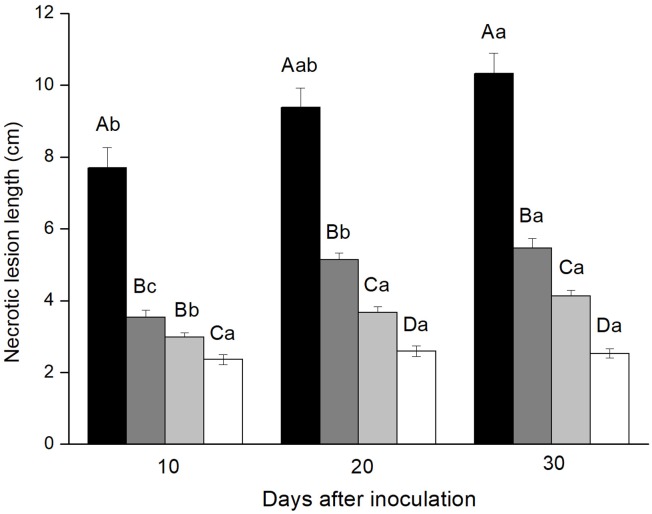
Necrotic lesion lengths in the phloem of *Pinus yunnanensis* following fungal inoculation or mechanical wounding (black bars: *Leptographium wushanense*, gray bars: *L. sinense*, light gray bars: *Ophiostoma canum*, white bars: agar control inoculation). The data are expressed as mean ± SE (*n* = 20). Different letters indicate significant differences between treatments at each time point (capital letters) or between time points for each treatment (lowercase letters), following Bonferroni or Dunnett’s T3 test at *P* = 0.05.

Lesion lengths increased significantly over time for *L. wushanense*: [*F*_(2,57)_ = 5.658, *P* < 0.01], *L. sinense* [*F*_(2,35.038)_ = 56.403, *p* < 0.01] and *O. canum* [*F*_(2,57)_ = 15.579, *p* < 0.001; Figure 1].

### Antioxidative Enzyme Activities in Phloem Lesions

**FIGURE 2:**
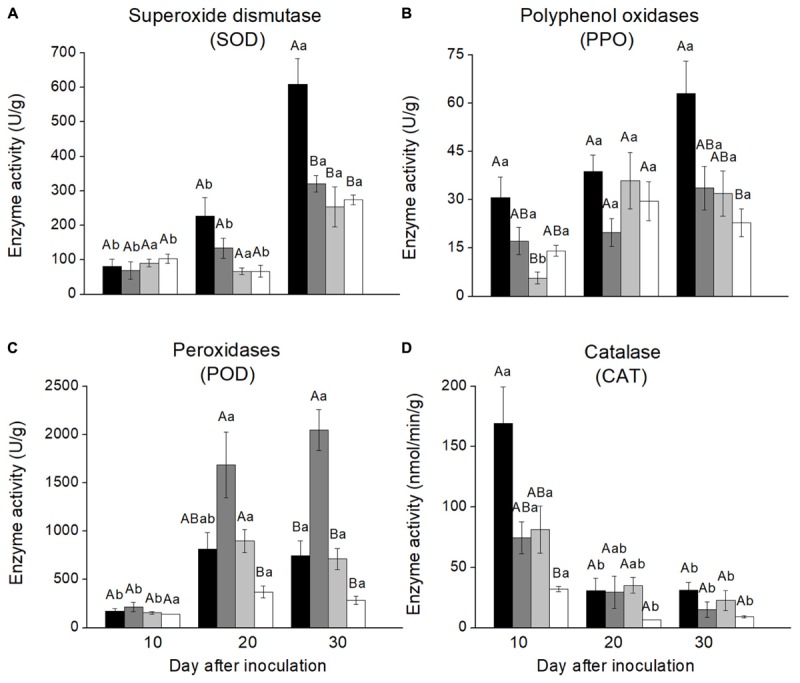
Antioxidative enzyme activities in the phloem reaction zones of *Pinus yunnanensis* following fungal inoculation or mechanical wounding (**A**, SOD; **B**, PPO; **C**, POD; **D**, CAT) (black bars: *Leptographium wushanense*, gray bars: *L. sinense*, light gray bars: *Ophiostoma canum*, white bars: agar control inoculation). The data are expressed as mean ± SE (*n* ≤ 10). Different letters indicate significant differences between treatments at each time point (capital letters) or between time points for each treatment (lowercase letters), following Bonferroni or Dunnett’s T3 test at *P* = 0.05.

Enzyme activities varied greatly among the four treatments and over time since inoculation. SOD activities in stem phloem inoculated with *L. wushanense* were significantly higher than for the three other treatments 30 days after inoculation [*F*_(3,9.131)_ = 6.382, *P* = 0.013]. There were no significant treatment differences at the other time points. SOD activities increased significantly over time since inoculation for all treatments except *O. canum* (Figure 2A and Supplementary Table S2).

PPO activities were more variable with few significant treatment differences, but PPO activities in phloem inoculated with *L. wushanense* were significantly higher than for *O. canum* 10 days after treatment [*F*_(3,8.765)_ = 6.770, *P* = 0.012]. The only treatment with a significant increase in PPO activities over time was *O. canum*, but it started out with very low activities 10 days after inoculation [*F*_(2,7.303)_ = 10.729, *P* = 0.007; Figure 2B and Supplementary Table S2].

POD activities in phloem inoculated with *L. sinense* were significantly higher than in the control 20 days after inoculation [*F*_(3,9.427)_ = 8.802, *P* = 0.004] and significantly higher than for the other treatments 30 days after inoculation [*F*_(3,11.580)_ = 24.407, *P* < 0.001]. Furthermore, POD activities increased significantly over time for all treatments except control (Figure 2C).

CAT activities showed a different temporal pattern than the other antioxidative enzymes, with CAT activities decreasing significantly over time for all four treatments (Figure 2). The only significant treatment difference was that *L. wushanense* had significantly higher CAT activities than the control 10 days after treatment [*F*_(3,9.420)_ = 10.663, *P* = 0.002 (C)].

### Monoterpene Concentrations in Phloem Lesions

**FIGURE 3:**
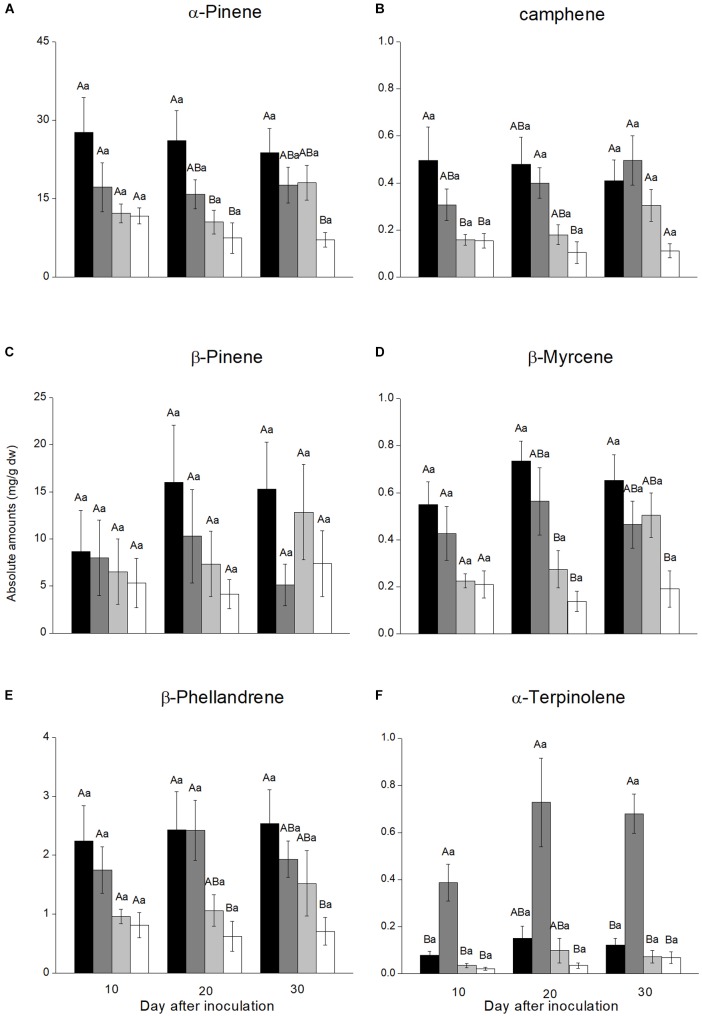
Monoterpene concentrations in necrotic lesions in the phloem of *Pinus yunnanensis* following fungal inoculation or mechanical wounding (**A**, α-pinene; **B**, camphene; **C**, β-pinene; **D**, myrcene; **E**, β-phellandrene; **F**, α-terpinolene) (black bars: *Leptographium wushanense*, gray bars: *L. sinense*, light gray bars: *Ophiostoma canum*, white bars: agar control inoculation). The data are expressed as mean ± SE (*n* ≤ 10). Different letters indicate significant differences between treatments at each time point (capital letters) or between time points for each treatment (lowercase letters), following Bonferroni or Dunnett’s T3 test at *P* = 0.05.

Six monoterpene hydrocarbons (α-pinene, camphene, β-pinene, myrcene, β-phellandrene, and α-terpinolene) were detected from phloem lesions induced by fungal and agar control inoculations (Figure 3). The most abundant monoterpenes in all treatments were α-pinene and β-pinene, which constituted more than 83.4% of total monoterpenes in most samples. There was no significant increase in monoterpene concentrations over time since inoculation for any treatment, although the absolute values increased gradually for most monoterpene-treatment combinations (Figure 3 and Supplementary Table S3).

Monoterpene concentrations varied between inoculation treatments. The two *Leptographium* species induced more monoterpene accumulation than *O. canum* and mechanical wounding. At the final sampling 30 days after inoculation, *L. wushanense* induced significantly higher concentrations of α-pinene [*F*_(3,13.193)_ = 7.172, *P* = 0.004], β-myrcene [*F*_(3,28)_ = 4.323, *P* = 0.013], and β-phellandrene [*F*_(3,__29)_ = 3.542, *P* = 0.027] compared to the control. In addition, *L. wushanense* also induced higher levels of camphene, β-pinene, and α-terpinolene than the control, but the differences were not statistically significant. Similarly, *L. sinense* induced a stronger accumulation of most monoterpenes than the control, but the increase was only significant for α-terpinolene [10^th^ day: *F*_(3,9.056)_ = 9.951, *P* = 0.003; 20^th^ day: *F*_(3,11.388)_ = 5.755, *P* = 0.012; 30^th^ day: *F*_(3,13.668)_ = 15.920, *P* < 0.001]. *Ophiostoma canum* induced higher absolute concentrations than the control for most monoterpenes, but the differences were not statistically significant for any monoterpene (Figure 3).

In addition to the differences observed at the final sampling time, one or both *Leptographium* species induced significantly higher concentrations than the control for α-pinene, camphene, β-myrcene, β-phellandrene, or α-terpinolene at 10 or 20 days after inoculation (Figure 3).

## Discussion

Blue-stain fungi are well-known associates of scolytid bark beetle species colonizing both hardwoods and conifers. Many blue-stain fungi are pathogenic to host trees, causing necrosis around natural infection points and experimental fungal inoculations. Because virulent fungi kill more host tissues and induce longer necrotic lesions than less virulent species, lesion length after fungal inoculation is used as a standard measure of fungal virulence (e.g., Krokene and Solheim, 1999; Solheim et al., 2001; Viiri et al., 2001; Fäldt et al., 2006). In the present study, inoculation of *L. wushanense* induced the longest lesions in Yunnan pine, followed by *L. sinense* and *O. canum*. This suggests that *L. wushanense* is the most virulent and *O. canum* the least virulent fungus of the three fungi we tested.

*Ophiostoma canum* is a common associate of *T. minor* and is weakly pathogenic to Scots pine (*P. sylvestris*) and Japanese red pine (*P. densiflora*) (Solheim et al., 2001; Masuya et al., 2003). We found that *O. canum* caused slightly, but significantly, longer lesions than mechanical wounding, suggesting that this fungus is weakly pathogenic also to Yunnan pine. The pathogenicity of *L. sinense* and *L. wushanense* was previously unknown. Our results show that they are moderately (*L. sinense*) or highly virulent (*L. wushanense*) to Yunnan pine. The presence of these pathogenic fungi in SW China might be a contributing factor to the destructiveness of *Tomicus* species in the area.

The two *Leptographium* species we studied were more virulent to our experimental trees than *O. canum* was. Blue-stain fungi in the genus *Leptographium* are primarily associated with bark beetles that attack live pine trees (Jacobs and Wingfield, 2001). These fungi seem to play a major role in decline diseases of several pine species, including red pine (*Pinus resinosa*) and other North American pines. Two pathogenic *Leptographium* species (*L. terebrantis* and *L. procerum*) introduced into red pine by the bark beetle species *Dendroctonus valens* and *Hylastes porculus* are thought to be a major cause of red pine decline in Midwestern United States (Klepzig et al., 1995). Another species, *L. wageneri*, cause black-stain root disease in several pines and other conifers and inflict considerable damage across western North America (Schweigkofler et al., 2005). In China, *L. yunnanense* vectored by *Tomicus* species are implicated in the ongoing decline of Yunnan pine (Liao, 2002). At the peak of the decline, *L. yunnanense* was isolated from 12.5% of the *T. yunnanenesis* individuals (Zhou et al., 2000). Our results suggest that also *L. sinense* and *L. wushanense*, which were isolated from *Tomicus* beetles tunneling in the relatively resistant *P. armandii* (Zhao and Längström, 2012), are virulent to pine trees and may contribute to the decline of Yunnan pine if they are transmitted to the tree by the bark beetles.

Production of antioxidative scavenging enzymes is part of the defense of many plants against biotic and abiotic stresses. Increased PPO activity in response to fungal infection in different plant species (Robert, 1966) is thought to increase plant resistance since PPO is involved in the formation of anti-fungal compounds and lignification process of host plants (Jung et al., 2004; Jaiti et al., 2007). Overexpression of PPO genes also increases resistance to herbivorous insects in tomato and hybrid aspen (Mahanil et al., 2008). Here, we found that inoculation of *L. wushanense* induced significantly higher PPO, SOD and CAT activities at one of the sampling times and *L. sinense* inoculation enhanced POD activity at the last two sampling times, indicating that these enzymes are involved in defense or repair responses of Yunnan pine against blue-stain fungi.

In addition to differences between inoculation treatments, there may be temporal differences in the induction of enzyme activities. After fungal inoculation of Yunnan pine, CAT activities were highest at the first sampling time, whereas SOD, POD, and PPO activities gradually increased over time and reached the highest level mostly at the last sampling time. This suggests that different antioxidative enzymes have different response speeds and that multiple sampling times are needed to get a complete picture of the enzyme activities. It is interesting to note that inoculation of *L. wushanense*, the most virulent fungus, induced the highest activities of SOD, PPO, and CAT activities, whereas the least virulent fungus *L. sinense* induced the highest POD activity. POD is involved in many physiological processes in plants, including responses to biotic and abiotic stress and lignin biosynthesis. These observations suggest that Yunnan pine activate different antioxidant enzymes depending on the nature of the attacking pathogens and the infestation stages.

Contrary to the increase observed after inoculation for the other antioxidant enzymes, CAT activity decreased significantly over time in all inoculation treatments (Figure 2). Very similar responses, with increased POD and SOD activities and decreased CAT activity, have been observed in liquorice (*Glycyrrhiza uralensis* Fisch) exposed to drought and salt stress (Pan et al., 2006). The underlying mechanism may be that SOD activity can increase concentrations of H_2_O_2_, which can react with superoxide to form hydroxyl radicals and cause a reduction in CAT activity (Pan et al., 2006). Different stress factors such as wounding, drought, pathogen infection and insect attack may induce similar responses (Nagy et al., 2004), and we speculated that the significant decline in CAT activity over time in our study might be due to the same mechanism.

Though small amounts of sesquiterpenes and diterpenes were found, monoterpenes were the main terpenoid class detected in our phloem samples. These 10-carbon terpenes are generally toxic to insects and may increase bark beetle mortality during host tree colonization (Gershenzon and Dudareva, 2007). Monoterpenes can also inhibit the growth of fungal pathogens (Cobb et al., 1968; Novak et al., 2014), such as the blue-stain fungi vectored by bark beetles. Terpene concentrations increase dramatically in the phloem of Norway spruce (*Picea abies*) following inoculation with the bark beetle-associated blue-stain fungus *Endoconidiophora polonica* (Zhao et al., 2011a). This terpene increase inhibits colonization by the spruce bark beetle (*Ips typographus*) in a dose-dependent manner (Zhao et al., 2011a). Norway spruce also produces monoterpenes with antifungal properties in response to infection by blue-stain fungi (Novak et al., 2014). Similarly, Scots pine trees produce more monoterpenes in the phloem following inoculation with the virulent fungus *L. wingfieldii* than with the less virulent species *O. canum* (Fäldt et al., 2006). We found the same pattern in the present study: *L. wushanense* and *L. sinense* induced a more intense monoterpene response than the less virulent *O. canum*. These observations suggest that Yunnan pine can distinguish between more and less virulent blue-stain fungi and activate appropriate inducible defenses according to the virulence of the attacker.

Conifers often have a very strong increase in terpene concentrations following fungal infection. For example, monoterpene levels in Scots pine phloem increase several 100-fold 4 weeks after inoculation with *L. wingfieldii* and *O. canum* (Fäldt et al., 2006) and terpene concentrations in Norway spruce phloem increases dramatically in response to *E. polonica* inoculation (Zhao et al., 2011a). Compared to Scots pine and Norway spruce the monoterpene response we observed in Yunnan pine to fungal inoculation was quite moderate, with small and mostly non-significant differences between sampling times. The different monoterpene response of Yunnan pine to other conifers may be a result of biological differences between both tree and fungal species In addition, the dry and warm weather in Yunnan may have impaired the terpene defense in Yunnan pine and made the trees more susceptibility to infection.

We inoculated our experimental trees with three fungal species and sampled the trees multiple times to compare fungal pathogenicity and disease symptoms over time. There is a possibility that the inoculations and sampling could have induced tree defense and thereby influence the results. However, many previous studies that have used a similar experimental design (Krokene et al., 1999; Fäldt et al., 2006; Zhao et al., 2018) have found negligible interference between treatments. Systemic priming or induction of tree defenses have been documented in Norway spruce (Krokene et al., 1999), but this require more time to become effective and more extensive and concentrated wounding than we used in our study. We thus trust that our experimental design have not interfered with our results. The considerable variation we observed within treatments may be due to the fact that we used naturally regenerated Yunnan pine trees of unknown genetic background. Extensive genetic differences between trees might explain why differences in terpene concentrations and antioxidant enzyme activities sometimes were unclear between treatments or time points.

Based on our observations we conclude that the three blue-stain fungi we studied are potentially pathogenic to Yunnan pine. The fact that *L. wushanense* and *L. sinense* elicited longer lesions and stronger monoterpene and antioxidant enzyme responses in Yunnan pine phloem than *O. canum* suggests that the two *Leptographium* species are more virulent to Yunnan pine than *O. canum*. So far, *L. wushanense* and *L. sinense* have only been isolated from restricted areas in SW China. However, they might be vectored by *Tomicus* beetles to Yunnan pine stands and cause serious damage in the future. Further studies are needed on their geographical distribution and pathogenicity mechanisms to better understand the risk they pose to Yunnan pine and to determine if they may facilitate tree-killing by *Tomicus* bark beetles.

## Author Contributions

HY and PC conceived the experiments. YP and JL designed and performed the experiments. Z-fY, MQ, HY, and PC contributed reagents, materials, and analysis tools. YP, TZ, and PK analyzed the data. TZ, YP, and PK wrote the paper. All authors revised the paper.

## Conflict of Interest Statement

The authors declare that the research was conducted in the absence of any commercial or financial relationships that could be construed as a potential conflict of interest.
