# Supplementary figures and images for: Bark Beetle-Associated Blue-Stain Fungi Increase Antioxidant Enzyme Activities and Monoterpene Concentrations in Pinus yunnanensis
Source: Front Plant Sci. 2018 Nov 27;9:1731. doi: 10.3389/fpls.2018.01731 (PMC6284243; doi:10.3389/fpls.2018.01731)

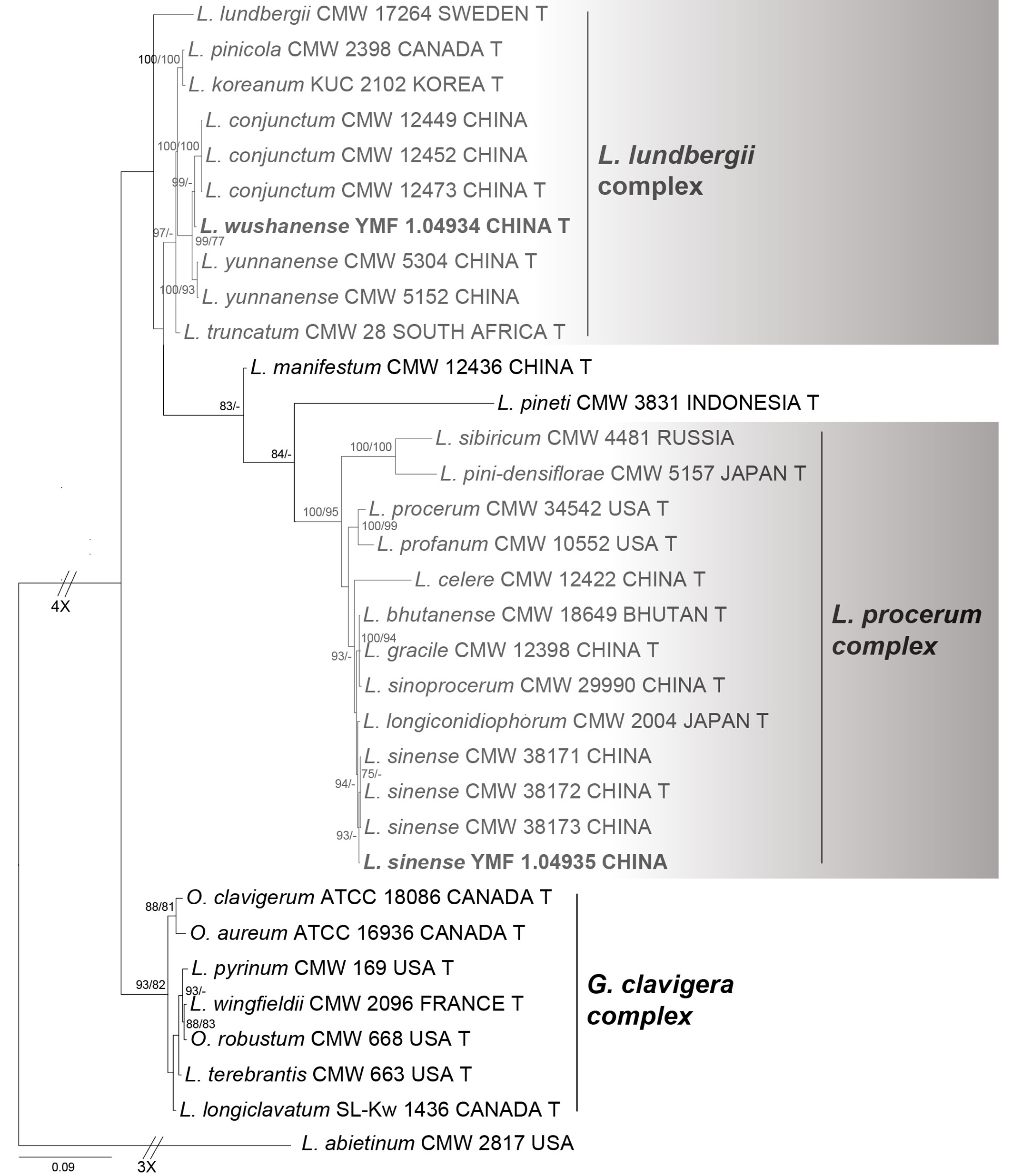

Supplement: Supplementary file 1 [file Image_1.JPEG]

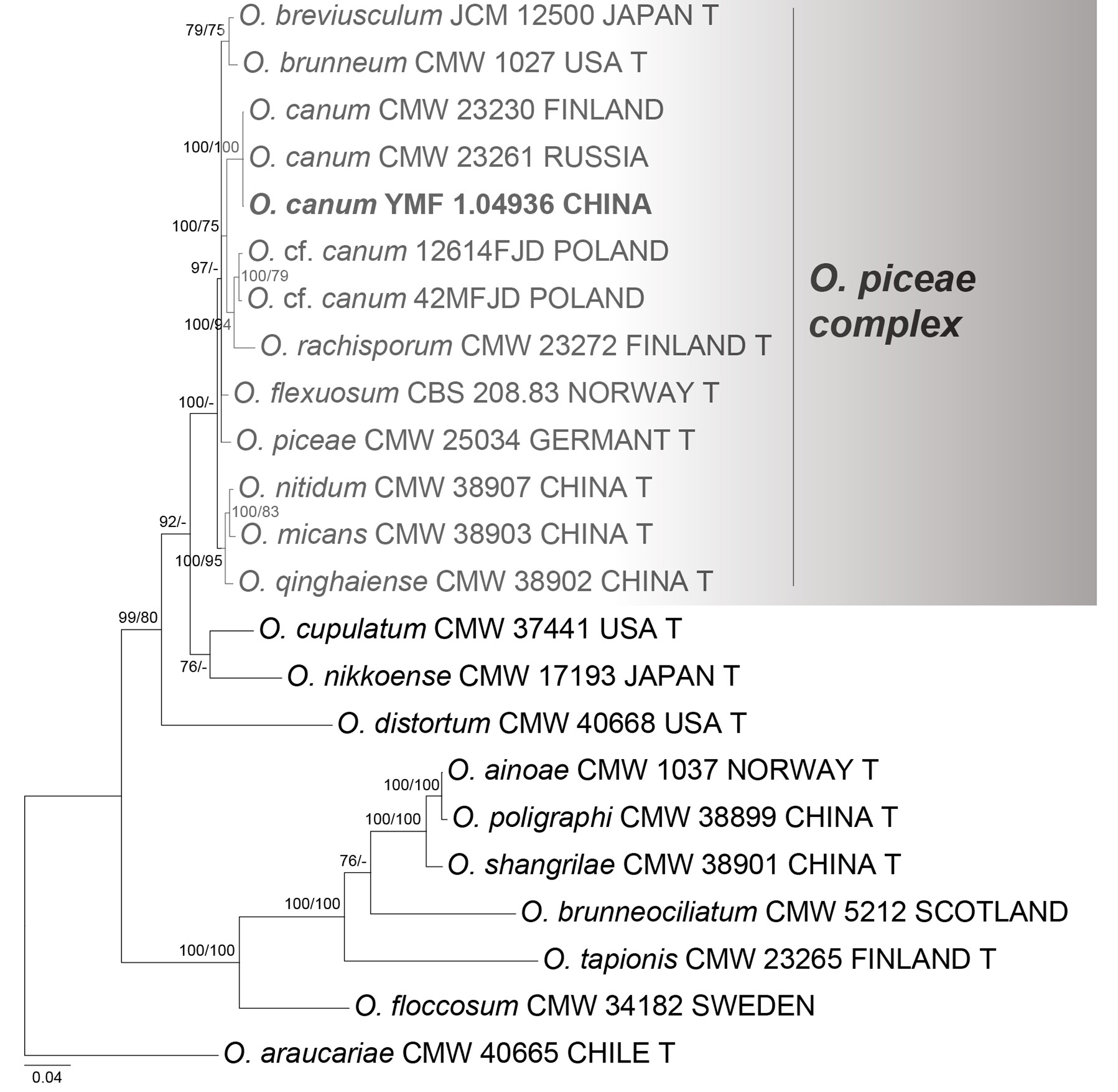

Supplement: Supplementary file 2 [file Image_2.JPEG]
