# Supplementary material for: Bark Beetle-Associated Blue-Stain Fungi Increase Antioxidant Enzyme Activities and Monoterpene Concentrations in Pinus yunnanensis
Source: Front Plant Sci. 2018 Nov 27;9:1731. doi: 10.3389/fpls.2018.01731 (PMC6284243; doi:10.3389/fpls.2018.01731)
Supplement: Supplementary file 3 [file Table_1.doc]

|  | Testing Method | Calculation formula |
| --- | --- | --- |
| Superoxide dismutases (SOD) | Test tube: 240 μL reagent ① + 510 μL reagent ② +36 μL reagent ③ + 90 μL sample extract + 180 μL reagent ④Control tube: 240 μL reagent ① + 510 μL reagent ② + 36 μL reagent ③ + 90 μL distilled water + 180 μL reagent ④  Absorbance (A) was recorded at 560 nm. | Inhibition percentage (IP) = (A _control tube_ - A _test tube_)/A _control tube_ × 100%  SOD (U/g) = [IP / (1- IP) × V_t_]/ (W × V_x_/V_tx_) × Sample dilution multiple  where V_t_ (total reaction volume): 1.026mL; V_x_ (volume of the sample): 0.09 mL; V_tx_ (volume of added extract): 1mL; W(the sample weight): 0.1 g. |
| Polyphenol oxidases (PPO) | Test tube: 600 μL reagent ① + 150 μL reagent ② + 150 μL sample extract  Control tube: 600 μL reagent ① + 150 μL reagent ② + 150 μL boiled enzyme extract  Absorbance (A) was recorded at 525 nm. | PPO (U/g) = (A _test tube_ - A _control tube_)× V_t_ /(W × V_x_/V_tx_)/0.01/T  where V_t_: 0.9mL; V_x_: 0.15 mL; V_tx_: 1mL; T (reaction time): 1min;  W: 0.1g |
| Peroxidases (POD) | 15 μL sample extract + 270μL distilled water + 520 μL reagent ① + 130 μL reagent ② + 134 μL reagent ③ were added to a 1 mL quartz cuvette  Absorbance (A) was recorded at 470 nm at 30 seconds (A_1_) and 1.5 min (A_2_). | POD (U/g)=(A_1_-A_2_) × V_t_/(W×V_x_/V_tx_)/0.01/T where V_t_: 1.07mL; V_x_: 0.015 mL; V_tx_ is the volume of added extract, 1 mL; T (reaction time): 1 min;  W: 0.1g. |
| Catalases (CAT) | 1 mL CAT working fluid and 35 μL sample extract were added to a 1 mL quartz cuvette and mixed well for 5 s.  Absorbance was recorded at 240 nm at the beginning (A_1_) and after 1 min (A_2_). | CAT (nmol/min/g) = [(A_1_-A_2_)×V_t_ /(ε × d10^9^] /(×V_x_/V_tx_)/T  Where Vt: 1.035 × 10^-3^ L; ε (H_2_O_2_ Molar extinction coefficient): 4.36 × 10^4^ L/mol/cm; V_x_: 0.035 mL; V_tx_: 1 mL; T: 1 min, W: 0.1 g |

Table S1 Methods used to determine antioxidative enzyme activities in *Pinus yunnanensis* phloem.
