# Supplementary material for: Bark Beetle-Associated Blue-Stain Fungi Increase Antioxidant Enzyme Activities and Monoterpene Concentrations in Pinus yunnanensis
Source: Front Plant Sci. 2018 Nov 27;9:1731. doi: 10.3389/fpls.2018.01731 (PMC6284243; doi:10.3389/fpls.2018.01731)
Supplement: Supplementary file 4 [file Table_2.doc]

Table S2 Interaction between treatments and sampling times on antioxidant enzyme activities in *Pinus yunnanensis*

| Antioxidant enzymes | Treatment | | | | Time | | | | Time * treatment | | | |
| --- | --- | --- | --- | --- | --- | --- | --- | --- | --- | --- | --- | --- |
|  | Treatment  df | Error  df | *F* | *P* | Time  df | Error  df | *F* | *P* | Interaction  df | Error df | *F* | *P* |
| Superoxide dismutases (SOD) | 3 | 65.01 | 4.088 | 0.010 | 1 | 0.55 | 6.168 | 0.380 | 3 | 65.01 | 12.839 | 0.000 |
| Polyphenol oxidases (PPO) | 3 | 107.95 | 0.356 | 0.785 | 1 | 161.86 | 12.101 | 0.001 | 3 | 163.56 | 0.497 | 0.685 |
| Peroxidases (POD) | 3 | 65.03 | 0.924 | 0.434 | 1 | 0.54 | 4.769 | 0.410 | 3 | 65.05 | 7.547 | 0.000 |
| Catalases (CAT) | 3 | 28.65 | 8.739 | 0.000 | 1 | 0.52 | 7.204 | 0.380 | 3 | 2.26 | 3.548 | 0.207 |
