# Supplementary material for: Bark Beetle-Associated Blue-Stain Fungi Increase Antioxidant Enzyme Activities and Monoterpene Concentrations in Pinus yunnanensis
Source: Front Plant Sci. 2018 Nov 27;9:1731. doi: 10.3389/fpls.2018.01731 (PMC6284243; doi:10.3389/fpls.2018.01731)
Supplement: Supplementary file 5 [file Table_3.doc]

Table S3 Interaction between treatments and sampling times on monoterpene concentrations in *Pinus yunnanensis*

| Monoterpenes | Treatment | | | | Time | | | | Time * treatment | | | |
| --- | --- | --- | --- | --- | --- | --- | --- | --- | --- | --- | --- | --- |
|  | Treatment df | Error  df | *F* | *P* | Time df | Error df | *F* | *P* | Interaction df | Error df | *F* | *P* |
| α-pinene | 3 | 132.82 | 2.979 | 0.034 | 1 | 132.82 | 0.038 | 0.846 | 3 | 132.82 | 1.092 | 0.355 |
| camphene | 3 | 80.00 | 3.115 | 0.031 | 1 | 80.00 | 1.056 | 0.307 | 3 | 80.00 | 1.947 | 0.129 |
| β-pinene | 3 | 82.00 | 0.344 | 0.794 | 1 | 82.00 | 0.432 | 0.513 | 3 | 82.00 | 0.412 | 0.745 |
| myrcene | 3 | 82.00 | 2.156 | 0.099 | 1 | 82.00 | 1.889 | 0.713 | 3 | 82.00 | 0.816 | 0.489 |
| β-phellandrene | 3 | 83.00 | 1.080 | 0.362 | 1 | 83.00 | 0.460 | 0.499 | 3 | 83.00 | 0.119 | 0.949 |
| α-terpinolene | 3 | 5.12 | 0.743 | 0.570 | 1 | 1.28 | 1.977 | 0.354 | 3 | 3.39 | 1.284 | 0.409 |
